# Supplementary material for: Identification of photoperiod-induced specific miRNAs in the adrenal glands of Sunite sheep (Ovis aries)
Source: Front Vet Sci. 2022 Jul 22;9:888207. doi: 10.3389/fvets.2022.888207 (PMC9354845; doi:10.3389/fvets.2022.888207)
Supplement: Supplementary file 1 [file Data_Sheet_1.ZIP › Supplementary file/miRDeep2 file.zip!/miRDeep2 file/Know_Structure/LP42A3/oar-mir-494.pdf]

miRBase precursor : oar-mir-494
Total read count : 61
oar-miR-494-5p read count : 0
oar-miR-494-3p read count : 59
remaining reads : 2

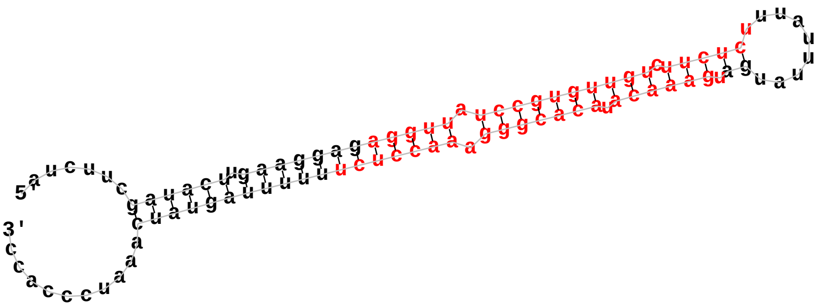

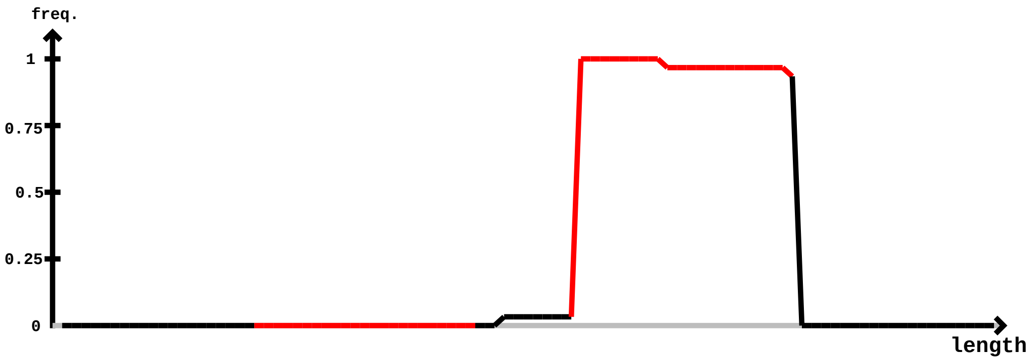


```
                                                   oar-miR-494-3p
                    oar-miR-494-5p
5'- aucuucgauacuugaaggagagguuauccguguugucuucucuuuauuuaugaugaaacauacacgggaaaccucuuuuuuaguaucaaaucccacc   -3'    exp
    ......(((((.(((((((((((.((((((((((.(((((.........)).)))))).))))))).))))))))))))))))).........    reads      mm         sample
    .............................................auuuaugaugaGacaua...................................    2          1          seq
    .....................................................ugaaacauacacgggaaaccuc......................    2          0          seq
    .....................................................ugaaacauacacgggaaaccucu.....................    57         0          seq
```
